# A Comparative Study of 0.0625% Bupivacaine-Fentanyl With 0.1% Ropivacaine-Fentanyl for Labor Epidural Analgesia

**DOI:** 10.7759/cureus.82681

**Published:** 2025-04-21

**Authors:** Chinthalapudi Mounika, Gabu Sujitha, Mrunalini P

**Affiliations:** 1 Department of Anaesthesia, NRI Medical College, Guntur, IND

**Keywords:** bupivacaine, epidural, fentanyl, labor analgesia, ropivacaine

## Abstract

Introduction: Labor pain triggers significant maternal stress, impacting both maternal and fetal well-being. Epidural analgesia is the gold standard for labor pain relief, offering effective analgesia with minimal side effects. This study compares the efficacy and safety of 0.0625% bupivacaine-fentanyl (BF) versus 0.1% ropivacaine-fentanyl (RF) in labor epidural analgesia.

Materials and methods: An observational study was conducted on 60 primiparous women in active labor, divided into two groups (n=30 each). Group BF received 0.0625% BF (2 mcg/ml), and Group RF received 0.1% RF (2 mcg/ml) via epidural infusion. Pain relief (Visual Analog Scale (VAS) score), motor block (modified Bromage score), need for top-ups, maternal satisfaction, mode of delivery, neonatal outcomes (Apgar scores), and adverse events were recorded. Data were analyzed using Epi Info (Centers for Disease Control and Prevention (CDC), Atlanta, Georgia), with p<0.05 considered significant.

Results: Both groups were demographically comparable. Group RF demonstrated superior analgesia with significantly lower VAS scores at 20 and 30 minutes (p=0.007 and p<0.001). Group BF required more top-ups and rescue analgesia. Motor blockade was significantly higher in Group BF (p=0.0074). Maternal satisfaction was better in Group RF (p=0.0005). Apgar scores and labor duration were comparable. No significant hemodynamic instability or adverse events were noted.

Conclusion: A combination of 0.1% RF provides superior analgesia with less motor block and higher maternal satisfaction compared to 0.0625% BF in labor epidural analgesia, without compromising neonatal outcomes.

## Introduction

Labor pain is one of the most intense experiences a woman can endure, and it triggers a significant maternal stress response. This stress can have negative consequences for both the mother and the fetus [1]. It can elevate blood pressure and heart rate, exacerbating the stress response and increasing the risk of maternal complications [2]. Maternal sympathetic activation due to labor pain can lead to irregular uterine contractions, potentially compromising fetal oxygenation and increasing the risk of maternal complications [3]. Moreover, maternal anxiety and pain are commonly associated with an increased preference for elective cesarean sections, as many women seek to avoid the pain of labor [4]. Consequently, effective pain relief during labor is crucial to reduce maternal and perinatal complications, as well as to minimize the need for cesarean deliveries induced by maternal distress.

Among the various pain management techniques available, neuraxial epidural analgesia has emerged as the most popular and effective modality for labor pain relief [5]. Epidural analgesia provides significant pain relief while allowing the mother to remain conscious and engaged during the delivery process. Furthermore, it reduces the risk of gastric aspiration, avoids the sedative effects associated with general anesthesia, and ensures that the mother can actively participate in the birth of her child [6]. As such, epidural analgesia has become a cornerstone in modern obstetric care, with the ability to significantly improve maternal comfort during labor [7]. Moreover, studies indicate its association with better neonatal outcomes, including reduced fetal distress [8].

The ideal analgesic for labor should provide long-lasting pain relief while minimizing motor impairment, limiting fetal drug transfer, and avoiding adverse effects on both the mother and the newborn [9]. Bupivacaine, a widely used local anesthetic for labor analgesia, has the advantages of long-lasting effects and limited placental transfer, making it a popular choice [10]. However, higher concentrations of bupivacaine can lead to side effects such as cardiotoxicity and motor blockade. To address these concerns, newer agents such as ropivacaine and levobupivacaine were developed. These agents have shown reduced cardiac and central nervous system toxicity while also offering less motor blockade compared to bupivacaine [11,12]. 

Ropivacaine, a less potent S-enantiomer of bupivacaine, produces a differential blockade, making it ideal for labor analgesia [13]. It provides effective pain relief with minimal motor block, allowing greater maternal mobility. Opioids such as fentanyl are often combined to enhance analgesia, reduce local anesthetic dose, and limit side effects [14,15]. These combinations allow for the reduction of anesthetic dosages, minimizing potential side effects while improving overall pain relief. A recent analysis also suggested that the combination of ropivacaine and fentanyl results in better pain control with fewer adverse effects on the neonate compared to other epidural regimens [8].

This study aims to compare the efficacy and safety of 0.0625% bupivacaine-fentanyl (BF) and 0.1% ropivacaine-fentanyl (RF) in labor epidural analgesia to determine the most effective regimen for both maternal and fetal well-being.

## Materials and methods

This observational study was conducted over 18 months, from 01 July 2022 to 31 January 2024, involving 60 primiparous women aged between 20 and 30 years with American Society of Anesthesiologists (ASA) grade II status in established labor. After obtaining approval from the Institutional Ethical Committee of NRI Medical College, Guntur, Andhra Pradesh, India (approval number: IEC PG30/Anaesth1/2021-22, dated 15^th^ June 2022) and written informed consent from the participants. Group BF (30 patients) received 0.0625% BF, while Group RF (30 patients) received 0.1% RF for labor epidural analgesia. The study aimed to evaluate the analgesic efficacy, maternal and neonatal outcomes, and adverse events associated with these two analgesic regimens.

Inclusion criteria were women with a singleton fetus in cephalic presentation and a cervical dilation of 3 cm, while exclusion criteria encompassed ASA Grade III or higher, anatomical spine deformities, coagulopathies, hemodynamic instability, severe aortic stenosis, and known allergies to local anesthetics. After confirming the onset of true labor and ensuring cervical dilation of 3 cm, baseline vital signs were recorded, and an IV line was established with Ringer’s lactate infusion at 100 ml/hr.

Epidural catheterization was performed under strict aseptic precautions at the L3-L4 interspace, followed by a test dose of 3 ml of 1% xylocaine with adrenaline to exclude intravascular placement. Group BF received an initial 10 ml bolus of 0.0625% bupivacaine, followed by a continuous infusion of 15 ml/hr of 0.0625% bupivacaine with 2 mcg/ml fentanyl. Group RF received an initial 10 ml bolus of 0.1% ropivacaine, followed by a continuous infusion of 15 ml/hr of 0.1% ropivacaine with 2 mcg/ml fentanyl. The local anesthetic solutions were diluted with normal saline to achieve the desired concentrations. The infusion continued until delivery.

The sample size was calculated to detect a minimum difference of 1.5 in Visual Analog Scale (VAS) scores between groups, assuming a standard deviation of two, 80% power, and a 5% significance level. Based on this, 28 participants were required per group; to account for dropouts, 30 were included in each group.

**Figure 1:**
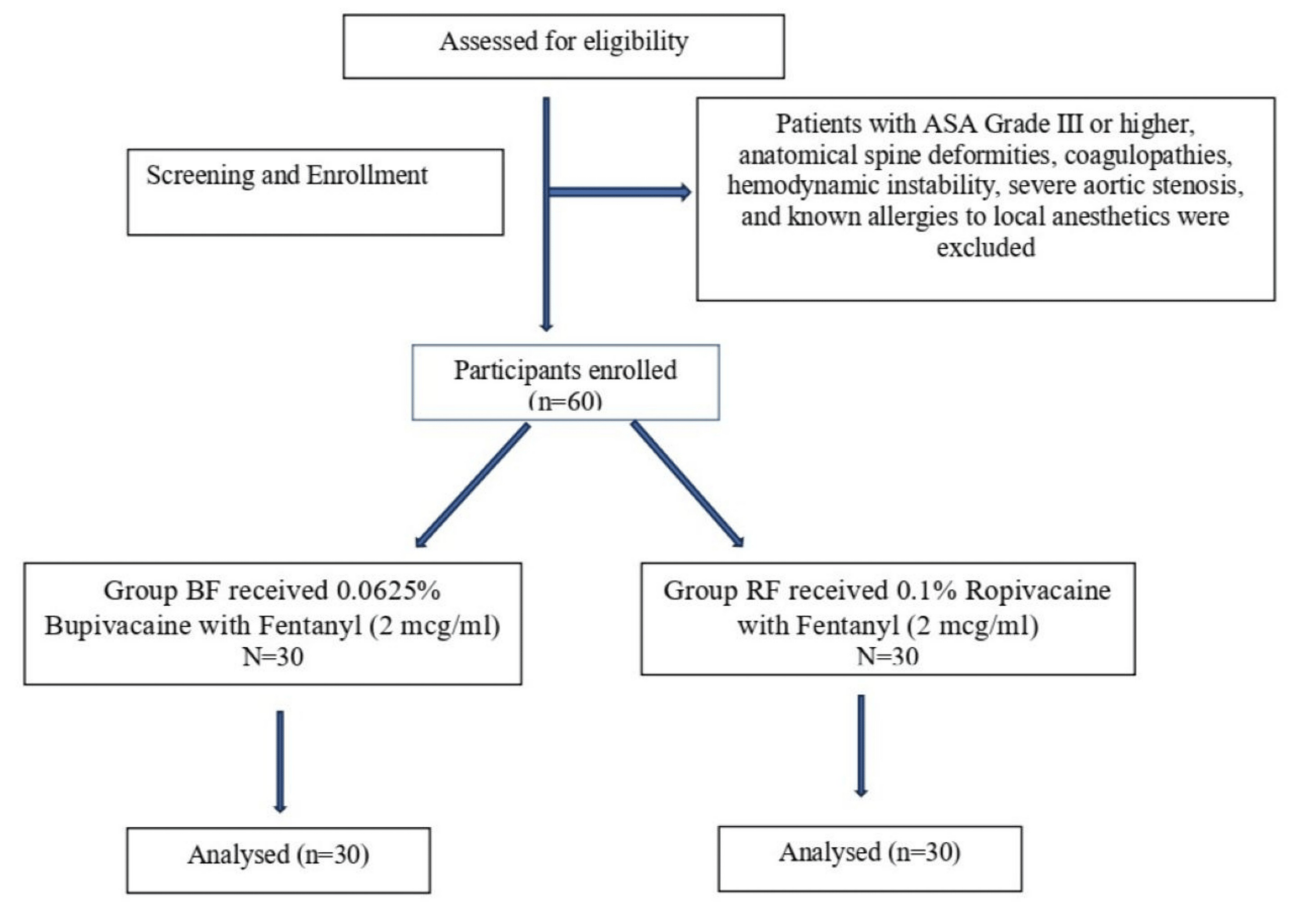
Consolidated Standards of Reporting Trials (CONSORT) flow diagram showcasing the disposition of the participants throughout the study ASA: American Society of Anesthesiologists; Group BF: bupivacaine with fentanyl group; Group RF: ropivacaine with fentanyl group

Pain intensity was evaluated using a verbal rating scale (0 to 10) at baseline and at 5, 10, 20, and 30 minutes after bolus administration. Sensory (pinprick testing) and motor blockade (modified Bromage score), blood pressure, heart rate, and oxygen saturation were closely monitored. Additional boluses of 5 mL of the respective study drug (either 0.0625% bupivacaine + fentanyl 2 mcg/mL or 0.1% ropivacaine + fentanyl 2 mcg/mL) were administered if the VAS score exceeded two. Maternal and neonatal outcomes, such as mode of delivery, duration of labor, Apgar scores, and maternal satisfaction (a four-point scale was used to measure satisfaction, where responses included 1: very dissatisfied; 2: dissatisfied; 3: satisfied; 4: very satisfied), were recorded. Adverse events, including hypotension and bradycardia, were managed according to standard protocols. Statistical analysis was performed using IBM SPSS Statistics software, version 26 (IBM Corp., Armonk, NY), with a p-value of <0.05 considered statistically significant (Figure 1).

## Results

The study compared the effectiveness and safety of two different labor epidural analgesia regimens: 0.0625% BF (Group BF) and 0.1% RF (Group RF). The demographic and baseline characteristics of the two groups were similar, with no significant differences in age (24.80 ± 3.37 years vs. 25.13 ± 3.56 years) and BMI (26.63 ± 1.90 kg/m² vs. 27.47 ± 1.66 kg/m²) (Table 1).

**Table 1:** Demographic and baseline characteristics of the participants Mean and SD were calculated for variables; a t-test was applied to derive the p-value. Group BF: bupivacaine with fentanyl group; Group RF: ropivacaine with fentanyl group

Variable	Group BF (n=30)	Group RF (n=30)	t-value	p-value
Age (years, Mean ± SD)	24.80 ± 3.37	25.13 ± 3.56	-0.37	0.7108
BMI (kg/m^2^, Mean ± SD)	26.63 ± 1.90	27.47 ± 1.66	-1.81	0.0755

The majority of patients in both groups experienced moderate pain at five and 10 minutes. However, at 20 and 30 minutes, a significantly higher number of patients in Group RF reported mild pain compared to Group BF (p = 0.007 and p < 0.001, respectively), indicating better pain control in Group RF over time (Table 2).

**Table 2:** Comparison of VAS pain score categories between Group BG and Group RF The VAS score was categorized as mild (0 to three), moderate (four to seven), or severe (eight to 10). Frequency and percentage were calculated for all variables. The chi-square test was applied to derive the p-value. VAS: Visual Analog Scale; Group BF: bupivacaine with fentanyl group; Group RF: ropivacaine with fentanyl group

Time point	VAS category	Group BF (n=30)	Group RF (n=30)	Chi-square value	p-value
5 minutes	Mild (0–3)	4 (13.3%)	5 (16.7%)	0.14	0.711
	Moderate (4–7)	26 (86.7%)	25 (83.3%)		
	Severe (8–10)	0 (0%)	0 (0%)		
10 minutes	Mild (0–3)	3 (10.0%)	4 (13.3%)	0.16	0.687
	Moderate (4–7)	27 (90.0%)	26 (86.7%)		
	Severe (8–10)	0 (0%)	0 (0%)		
20 minutes	Mild (0–3)	5 (16.7%)	14 (46.7%)	7.16	0.007
	Moderate (4–7)	25 (83.3%)	16 (53.3%)		
	Severe (8–10)	0 (0%)	0 (0%)		
30 minutes	Mild (0–3)	1 (3.3%)	17 (56.7%)	20.51	<0.001
	Moderate (4–7)	29 (96.7%)	13 (43.3%)		
	Severe (8–10)	0 (0%)	0 (0%)		

In terms of analgesic efficacy, the BF group required more top-up doses (1.13 ± 0.90 vs. 0.50 ± 0.57, p=0.0019). The median (interquartile range (IQR)) number of rescue analgesia doses used was similar between the groups, 1 (0-2) in both Group BF and Group RF; however, the difference was still statistically significant (p = 0.0267). The modified Bromage score (maximum observed) was also higher in the BF group (2.80 ± 1.06 vs. 2.10 ± 0.85, p=0.0074), indicating a greater motor block. At 30 minutes, the maximum level of sensory block was comparable between the two groups, with Group BF achieving sensory block at the T4, T5, and T6 levels in 14, nine, and seven participants, respectively, while Group RF had 13, 11, and six participants, respectively, at the same levels. Statistical analysis showed no significant difference in the distribution of sensory block between the two groups (p=0.8547) (Table 3).

**Table 3:** Comparison of analgesic efficacy between groups A t-test was applied to derive the p-value. Group BF: bupivacaine with fentanyl group; Group RF: ropivacaine with fentanyl group; IQR: interquartile range

Parameter	Group BF (n=30)	Group RF (n=30)	t-value	p-value
Top-up doses used (mean ± SD)	1.13 ± 0.90	0.50 ± 0.57	3.25	0.0019
Number of rescue analgesia doses used (median (IQR))	1 (0-2)	1 (0-2)	1.12	0.0267
Modified Bromage score (maximum observed) (mean ± SD)	2.80 ± 1.06	2.10 ± 0.85	-2.77	0.0074

Regarding maternal and neonatal outcomes, there were no significant differences between the groups. Both groups had 100% normal vaginal deliveries. The Apgar scores at one minute (9.13 ± 0.78 vs. 8.83 ± 0.79, p=0.1437) and at five minutes (9.90 ± 0.78 vs. 9.60 ± 0.79, p=0.24) were similar between the two groups. Maternal satisfaction scores were significantly higher in Group RF (2.80 ± 1.06 vs. 3.57 ± 0.50, p=0.0005) (Table 4).

**Table 4:** Maternal and neonatal outcomes Mean and SD were calculated. A t-test was applied to derive the p-value. Group BF: bupivacaine with fentanyl group; Group RF: ropivacaine with fentanyl group

Outcome	Group BF (n=30)	Group RF (n=30)	t-value	p-value
Apgar score at 1 minute (mean ± SD)	9.13 ± 0.78	8.83 ± 0.79	1.48	0.1437
Apgar score at 5 minutes (mean ± SD)	9.90 ± 0.78	9.60 ± 0.79	0.41	0.24
Maternal satisfaction Score (mean ± SD)	2.80 ± 1.06	3.57 ± 0.50	-3.66	0.0005

The duration of labor was comparable between the two groups (244.5 ± 8.86 minutes vs. 249.0 ± 7.27 minutes, p=0.51), and there were no significant differences in mean arterial pressure (MAP) at five, 10, or 30 minutes (Table 5).

**Table 5:** Labor duration and hemodynamic parameters Mean and SD were calculated. A t-test was applied to derive the p-value. Group BF: bupivacaine with fentanyl group; Group RF: ropivacaine with fentanyl group; MAP: mean arterial pressure

Parameters (mean ± SD)	Group BF (n=30)	Group RF (n=30)	t-value	p-value
Duration of labor (minutes)	244.5 ± 8.86	249.0 ± 7.27	0.95	0.51
MAP at 5 minutes	86.73 ± 8.86	85.30 ± 7.27	0.68	0.4961
MAP at 10 minutes	84.73 ± 9.01	87.53 ± 8.48	-1.24	0.2201
MAP at 30 minutes	80.53 ± 6.15	80.90 ± 6.21	-0.23	0.8190

## Discussion

The present study aimed to compare the efficacy of RF and BF for labor analgesia in 60 women admitted to a tertiary care center. Our findings revealed that both ropivacaine and bupivacaine provided effective analgesia, with no significant differences observed in demographic factors such as age, BMI, and gestational age, ensuring the comparability of the two groups at baseline. These results align with previous studies by Kulkarni et al. and Chora et al., which also found no significant differences in demographic characteristics between the RF and BF groups. The homogeneity of baseline characteristics further supports the internal validity of our study [15, 16].

In terms of analgesic efficacy, our study demonstrated that RF provided superior results compared to BF, with significant differences in the VAS scores observed at 20 and 30 minutes post-administration. The RF group showed lower VAS scores at these time points, indicating more effective pain relief. These findings are consistent with previous studies, such as Kulkarni et al. and Shivani et al., where ropivacaine also yielded better analgesic outcomes compared to bupivacaine [15, 17]. Additionally, our results showed that fewer top-up doses and rescue analgesia were required in the RF group, reinforcing the higher efficacy of ropivacaine for labor analgesia. These outcomes are in agreement with Chora et al., who also reported longer duration of analgesia with ropivacaine and fewer top-up doses compared to bupivacaine [16].

Regarding maternal satisfaction and neonatal outcomes, our study found that the RF group had a significantly higher maternal satisfaction score compared to the BF group, further supporting the greater efficacy and satisfaction associated with ropivacaine. This is consistent with studies by Bhatia et al. and Chora et al., where maternal satisfaction was notably higher in the ropivacaine groups [16, 18]. Moreover, no significant differences were observed in Apgar scores between the two groups, suggesting that both RF and BF provided safe analgesia without negatively impacting neonatal outcomes. This aligns with previous research, which also reported no adverse effects on neonatal health when either ropivacaine or bupivacaine was used for labor analgesia [15].

Finally, while there was a significant difference in the degree of motor blockade, with the BF group exhibiting more motor block than the RF group, no adverse clinical outcomes, such as cesarean sections or postpartum hemorrhage, were observed in either group. These findings corroborate those of Kulkarni et al. and Bhatia et al., who similarly reported no significant adverse outcomes related to motor blockade [15, 18]. Furthermore, our study found no significant differences in hemodynamic parameters, such as MAP and heart rate, between the two groups, indicating that both anesthetic regimens were well-tolerated and did not significantly affect maternal hemodynamics, consistent with the results of Kulkarni et al. [15]. Overall, this study reinforces that RF is a more efficacious and satisfactory choice for labor analgesia compared to BF, with no significant adverse effects on maternal or neonatal health.

Limitations

The present study, while providing valuable insights into the comparative efficacy of 0.0625% BF and 0.1% RF for labor epidural analgesia, has certain limitations that must be acknowledged. The sample size was relatively small and limited to a single tertiary care center, which may affect the generalizability of the results to broader populations with diverse demographic and clinical profiles. Additionally, the short duration of follow-up limited the ability to assess long-term maternal and neonatal outcomes or late-onset complications. Furthermore, the study did not evaluate the pharmacoeconomic aspects of the two regimens, which could be relevant in resource-limited settings. These limitations highlight the need for larger, multicentric studies to validate and extend the current findings.

## Conclusions

This study demonstrated that both epidural RF and BF effectively maintained hemodynamic stability, with no adverse effects or conversions to cesarean section. However, RF showed superior efficacy in sensory and motor blockade, as well as analgesia, leading to enhanced maternal satisfaction. Both regimens were safe, and all patients were discharged in stable condition. Based on these findings, we recommend RF as the preferred choice for epidural analgesia in patients scheduled for normal labor, owing to its superior efficacy compared to BF.
